# Alcohol and risk of admission to hospital for unintentional cutting or piercing injuries at home: a population-based case-crossover study

**DOI:** 10.1186/1471-2458-11-852

**Published:** 2011-11-09

**Authors:** Simon Thornley, Bridget Kool, Elizabeth Robinson, Roger Marshall, Gordon S Smith, Shanthi Ameratunga

**Affiliations:** 1 Section of Epidemiology & Biostatistics, School of Population Health, University of Auckland, Private Bag 92019, Auckland 1142, New Zealand; 2 Department of Epidemiology & Public Health, University of Maryland School of Medicine, National Study Center for Trauma & EMS, Baltimore, MD, USA

**Keywords:** Alcohol drinking, Cross-over studies, Cutting and piercing, Wounds and injuries

## Abstract

**Background:**

Cutting and piercing injuries are among the leading causes of unintentional injury morbidity in developed countries. In New Zealand, cutting and piercing are second only to falls as the most frequent cause of unintentional home injuries resulting in admissions to hospital among people aged 20 to 64 years. Alcohol intake is known to be associated with many other types of injury. We used a case-crossover study to investigate the role of acute alcohol use (i.e., drinking during the previous 6 h) in unintentional cutting or piercing injuries at home.

**Methods:**

A population-based case-crossover study was conducted. We identified all people aged 20 to 64 years, resident in one of three regions of the country (Greater Auckland, Waikato and Otago), who were admitted to public hospital within 48 h of an unintentional non-occupational cutting or piercing injury sustained at home (theirs or another's) from August 2008 to December 2009. The main exposure of interest was use of alcohol in the 6-hour period before the injury occurred and the corresponding time intervals 24 h before, and 1 week before, the injury. Other information was collected on known and potential confounders. Information was obtained during face-to-face interviews with cases, and through review of their medical charts.

**Results:**

Of the 356 participants, 71% were male, and a third sustained injuries from contact with glass. After adjustment for other paired exposures, the odds ratio for injury after consuming 1 to 3 standard drinks of alcohol during the 6-hour period before the injury (compared to the day before), compared to none, was 1.77 (95% confidence interval 0.84 to 3.74), and for four or more drinks was 8.68 (95% confidence interval 3.11 to 24.3). Smokers had higher alcohol-related risks than non-smokers.

**Conclusions:**

Alcohol consumption increases the odds of unintentional cutting or piercing injury occurring at home and this risk increases with higher levels of drinking.

## Background

Cutting and piercing injuries are among the leading causes of unintentional injury morbidity in developed countries [1,2]. In New Zealand, cutting and piercing are second only to falls as the most frequent cause of unintentional injuries among people aged 20 to 64 years that result in admission to hospital [3]. Almost 30% of cutting and piercing injuries in this age group that result in admission to hospital in New Zealand, occur at home [4]. As many as 20% of young and middle-aged adults admitted to hospital as a result of a cutting or piercing injuries may have consumed alcohol in the 6 h preceding injury [5]. Based on its association with many other types of injury including cutting and piercing injuries, alcohol intake could be an important target for intervention [6-8]. Injuries are recognised as the leading cause of alcohol attributable deaths globally (37.8% in 2004) [9]. While the harmful influence of alcohol is likely to be mediated in part through predictable cognitive and psychomotor effects-such as reaction time, cognitive processing, coordination and vigilance [10], the contribution of alcohol use to cutting and piercing injury is unclear.

The case-crossover research design has been shown to be well suited to study the influence of transient exposures that occur intermittently [11]. Previous case-crossover studies for injuries have investigated the influence of alcohol [12-15], cannabis use [16], anger [17], cell phone use [18], sleepiness [19,20], and other transient risk factors [21-23]. This method has also helped identify causes of study work-place hand injuries (some of which were due to cutting and piercing) [24-27] but we found no similar studies in the home setting.

We used a case-crossover study to investigate the role of acute alcohol use (drinking during the previous 6 h) in the occurrence of unintentional cutting or piercing injuries at home. We restricted ourselves to home injuries because this study was funded as part of a program studying home injuries and because exposures were more likely to be similar than those occurring outside the home.

## Methods

We identified all people aged 20 to 64 years, resident in one of three regions of the country (Greater Auckland, Waikato and Otago), who were admitted to public hospital within 48 h of an unintentional non-occupational cutting or piercing injury sustained at home (theirs or another's) from August 2008 to December 2009. In New Zealand, about 97% of acute injury admissions are to public hospitals [28]. Admission registers of the five recruiting hospitals (North Shore, Auckland City, Middlemore, Waikato, and Dunedin) were reviewed by study nurses to identify potential cases meeting the inclusion criteria.

Patients who provided written informed consent were interviewed face-to-face by research nurses using a structured questionnaire, which took about 30 min to complete. Interviews were conducted as soon after the injury as practically possible. The main alcohol related exposure of interest was acute alcohol intake (converted to standard 12 g alcohol units) in the 6 h immediately prior to injury. As this was a case-crossover study we obtained the same information on alcohol intake in two control periods at the same time of day for the 6 h reference time period: the previous day and the same day the previous week.

Other information was collected on known and potential confounders including: sociodemographic characteristics, medical conditions, smoking status, prescription drug use, acute marijuana and other illicit drug use (within 3 h of injury), acute sleep deprivation (less than 6 h sleep during the previous 24-hours), usual marijuana and other illicit drug use (at least weekly), and usual alcohol use indicative of hazardous or harmful drinking using the standardised Alcohol Use Disorders Identification Test (AUDIT) scale [29]. The AUDIT score is categorised into four risk levels: low risk (score 0-7), hazardous drinking (8-15), severe hazardous drinking (16-19), and probable dependence ≥ 20). For potential confounders that vary with time (acute recreational drug use and sleep deprivation) the same information was collected for the two control periods.

The power associated with the expected sample size (*n *= 313; 38 discordant individuals), was estimated using information from a case-crossover study which investigated the role of alcohol consumption in injury in the United States [13]. In this study, 12% of participants had discordant exposure in case and control exposure periods when "none" to "any" alcohol use was compared [13]. From this information, and assuming a relative risk (RR) of 3, the sample size was expected to achieve 80% power at the 0.05 level of significance [30].

The effect of alcohol was examined using the case-crossover, matched-pair-interval approach [11]. We categorised alcohol consumption in two different ways. First, any drinking was compared to no drinking during the case and control periods. In addition, to investigate dose-response effects, no alcohol intake was contrasted to 1 to 3 drinks, or 4 or more.

Effects of binary exposure variables were analysed using discordant pairs ratios, comparing each control period separately against the event period. We also used conditional logistic regression modelling to estimate exposure effects taking account of both control periods in a single model. The model also allowed adjustment for confounding variables. Marijuana and sleep deprivation were considered to be key confounding variables. If either alcohol, marijuana or sleep deprivation were missing from one exposure period, the information for this time period was omitted from the analysis. Other potential confounders were included in models if these responded in an incremental change in the effect of alcohol of 10% or more [31]. Analyses explored whether smoking, socio-economic status, and other modified the effect of acute alcohol use by including interaction terms and subgroup analyses.

All analyses were conducted using the R-project statistical software with the 'survival' package, using the 'clogit' procedure for conditional logistic regression [31].

The study was approved by the national ethics committee (MEC/08/13/EXP), and the relevant institutional and Māori research boards of the five recruiting hospitals. The study was carried out in compliance with the Helsinki Declaration http://www.wma.net/e/policy/b3.htm.

## Results

In total, 456 cases met the inclusion criteria, of whom 10 had insufficient English language skills to complete the interview (2%) and a further 90 (20%) were excluded due to being missed at presentation (*n *= 20) or because they declined to participate (*n *= 70). Non-respondents had a similar gender distribution to respondents, but were more likely to identify as either Māori (33.0% cf. 17.4%) or Pacific ethnicity (26.0% cf. 10.7%), and were more likely to be younger (20-39 years 64.5% cf. 47.8%). The median age of the remaining 356 participants was 40 years (Interquartile-range 28 to 51). Contact with glass (31%, *n *= 111), powered tools or machinery (29%, *n *= 103) accounted for the most injuries (Table 1). Blood alcohol concentration testing was only performed in 18 cases (5.1%), 16 of these were positive.

**Table 1:** Study population characteristics (*n *= 356)

Characteristic	No. of subjects	%
Gender		

Male	252	70.8

Female	104	29.2

Age category (in years)		

20 to 29	95	26.7

30 to 39	75	21.1

40 to 49	81	22.8

50 to 59	73	20.5

60 to 65	32	9.0

Ethnic group		

Māori	62	17.4

NZ European	193	54.2

Other	63	17.7

Pacific	38	10.7

Highest qualification		

None	102	28.7

School certificate	43	12.1

Overseas secondary school	15	4.2

Sixth form cert./Bursary	29	8.1

Trade certificate	87	24.4

Tertiary degree	66	18.5

Missing/Refused	14	3.9

In paid employment		

Yes	244	68.5

No	109	30.6

Missing/Refused	3	0.8

Object involved in injury		

Glass	111	31.2

Powered hand tools/machinery	103	28.9

Foreign body	39	11.0

Knife/sword/dagger	39	11.0

Non-powered hand tool	35	9.8

Lawnmower	28	7.9

Missing/Unknown	1	0.3

AUDIT score		

0-7 low risk	242	68.0

8-15 hazardous drinking	71	19.9

16-19 severe hazardous drinking	13	3.7

≥ 20 probable dependence	14	3.9

Missing/Refused	16	4.5

Acute marijuana use (3 h before injury)		

Yes	8	2.2

No	345	96.9

Missing/Refused	3	0.8

Usual marijuana use (at least weekly)		

Yes	39	11.0

No	315	88.5

Missing/Refused	2	0.6

Other recreational drug use (at least weekly)		

Yes	25	7.0

No	329	92.4

Current smoker		

Yes	104	29.2

No	252	70.8

Acute sleep deprivation < 6 h in previous 24 h)		

Yes	251	70.5

No	21	5.9

Missing/unknown	84	23.6

The majority (68%, *n *= 242) of participants reported a long term pattern of alcohol use consistent with a low risk of hazardous or dependent drinking (AUDIT score < 8), few (4%, *n *= 14) reported regular use indicative of alcohol dependency (AUDIT score ≥ 20), and a further 5% (*n *= 16) of individuals refused to respond to these questions (Table 1).

Overall, for the 1068 exposure periods of interest (alcohol, marijuana and sleep deprivation), 89 (8.3%) were dropped in multivariate analyses due to missing information in one of the three paired exposures (alcohol use, sleep deprivation or marijuana use). For the acute alcohol use data, missing information due to refusal to respond or difficulty with recall in the injury or control periods, resulted in complete paired entries for 345/356 participants for the 'day before', and 311/356 participants for 'a week ago' (Table 2). Although many individuals could not recall the duration of the sleep they had in the 24 h before the injury (*n *= 84, 23.6%), fewer instances of missing information were recorded for this exposure in the 'day-before' control period (*n *= 48, 13.5%), compared to the 'week-before' control period (*n *= 84, 23.6%). The majority of subjects reported no alcohol use in the 6 h before injury, or in the corresponding period either the day before (257/345) or the week before (253/320) the injury.

**Table 2:** Alcohol consumption in the 6 h preceding injury and the control periods

	Alcohol consumption in the 6 h before injury			
	
Alcohol consumption during control periods	0 drinks	1 to 3 drinks	4 or more drinks	Total (%)
Day before (*n *= 345)				

0 drinks	257	22	29	308 (89.3)

1 to 3 drinks	10	6	3	19 (5.5)

4 or more drinks	6	2	10	18 (5.2)

Total	273	30	42	345 (100)

Week before (*n *= 320)				

0 drinks	253	18	19	290 (93.2)

1 to 3 drinks	10	6	3	19 (6.1)

4 or more drinks	2	0	9	2 (0.6)

Total	265	24	22	320 (100)

After adjustment for other paired exposures, alcohol consumption in the 6 h period before injury was positively associated with cutting and piercing injury, when both 'day before' and 'week before' control periods compared 'any drinking' to 'no drinking', with a threefold elevated odds of injury in the former group (Table 3). A dose-response effect was evident, when the association between the intake of 1 to 3 drinks and 4 or more drinks with cutting and piercing injuries were contrasted. The adjusted odds ratios (OR) for 4 or more drinks was more than 2.5 times that for 1 to 3 drinks (OR 8.68; 95% CI: 3.11, 24.3 vs. OR 1.77; 95% CI: 0.84, 3.74).

**Table 3:** Summary of crude and adjusted effect measures for unintentional cutting or piercing injuries

	Control Periods					
		
	'Day before'		'Week before'			
		
Exposure	Crude OR	95% CI^†^	Crude OR	95% CI	Adjusted* OR	95% CI
Alcohol ≥ 1 vs. 0 standard drinks)	3.19	1.79, 5.99	3.08	1.57, 6.49	3.28	1.78, 6.06

Alcohol (1 to 3 vs. 0 standard drinks)	2.20	1.00, 5.20	1.80	0.79, 4.36	1.77	0.84, 3.74

Alcohol ≥ 4 vs. 0 standard drinks)	4.83	1.97, 14.2	9.50	2.29, 8.41	8.68	3.11, 24.3

OR, odds ratio

Crude OR calculated as discordant pairs. For example, 3.19 = (22 + 29)/(10 + 6) from Table 2

*From a conditional logistic regression, accounting for both control periods ('day before' and 'week before') and adjusted for marijuana use, and sleep deprivation

^†^CI, confidence interval

We evaluated whether the association between cutting or piercing injury and acute alcohol use across all control periods was modified by the participants' smoking status. There was a strong smoking-alcohol interaction; analysis of smokers and non-smokers gave an adjusted odds ratio for injury after more than 1 alcoholic drink among current smokers was 15.5 (95% CI: 3.18, 75.8), compared to 1.69 (95% CI: 0.79, 3.62) for non-smokers. Smokers reported having drunk larger amounts at the time of injury with 9.8% of smokers reporting 4 or more drinks (8.8%; 7 or more), and non-smokers 3.3% (1.6%; 7 or more).

No interactions were observed between acute alcohol use and age, gender, education, fatigue, usual pattern of alcohol use, or recreational drug use and the outcome risk of cutting or piercing injury, suggesting that all drinkers were at higher risk of cutting and piercing injuries after consuming alcohol, not just those with a high risk of hazardous or dependent drinking.

## Discussion

These findings indicate that acute alcohol use (within 6 h of injury) is associated with hospital treatment for unintentional cutting or piercing injuries at home, among young and middle-aged adults. There is evidence of a dose-response relationship with the adjusted odds ratio for 4 or more drinks being considerably higher than that for 1 to 3 drinks, relative to no drinks (8.68 compared with 1.77, respectively). Smoking status modified the effect of alcohol on injury, so that the excess odds of alcohol exposure were much higher among smokers than non-smokers.

The strengths of this population-based study include the relatively high response rate of around 80%. The study base-Greater Auckland, Waikato and Otago regions--covers more than 60% of the total New Zealand population aged 20 to 64 years, and includes both rural and urban environments. The findings, however, need to be considered in light of several limitations. Although the study was designed to be population-based, the higher proportion of individuals of Māori and Pacific ethnicity among non-respondents has introduced a degree of selection bias. The study relied on self-reported data for capturing acute exposures and lifestyle factors and blood alcohol concentration (BAC) was only measured in 5.1% of cases. The accuracy of the information, provided by participants, limits the credibility of our reported effect measures. Actual intake may be underestimated, as has been found with other self report measures, due to reluctance to admit to consumption, or simply poor recall particularly for the period 1 week before the injury occurred [32,33]. Increased missing information for the week before (over the day before) provides evidence for this effect (Table 2). In subjects with high levels of alcohol use (either before the event or who reported habitual high levels of intake), reported number of units of alcohol consumed during specific periods is unlikely to be accurate [34]. Furthermore, as suggested by the wide confidence intervals around estimates for some of the effect modification analyses (e.g. AUDIT category ≥ 20, and smokers), the study was too small to allow precise subgroup analyses.

The prevalence of hazardous drinking as measured by the AUDIT score (≥ 8) was 32.0%, higher than the 21% of New Zealand adults who identified as having a hazardous drinking pattern in the most recent New Zealand Health Survey (2006/07) [35]. Our findings were, however, similar to the proportion of 25 to 59 year olds who had a moderate to severe injury as a result of an unintentional fall at home (24.5%) [36]. Twenty-nine percent of our participants identified as 'current smokers'. This proportion is higher than New Zealand national estimates which indicate that 22% of adults (15 to 64 years) are current smokers [37], but it is lower than a US study of moderate to severely injured adult (18 to 65 years) trauma patients (Injury Severity Score > 20), who were admitted to hospital of whom 47.7% were current smokers [38].

Given the study entry criteria, it is not possible to determine how generalisable the findings are to cutting and piercing injuries that are fatal, do not result in hospitalisation, or occur in settings outside the home, such as workplace or recreational environments.

In case-crossover studies, it is important to select control periods that are sufficiently distant in time from the case period to limit the correlation between the two periods [39]. Our selection of 24 h before and 1 week before are consistent with other case-crossover studies which investigate the role of acute alcohol on injury risk [13,40]. The 'hangover' or 'residual alcohol' effect in which fatigue may play a role, has been identified as a potential risk factor in previous injury studies [41-45]. The selection of the first control period (the same 6 h in the 24 h prior to the injury occurring) may have limited our ability to assess this phenomenon. However, the point estimates and confidence intervals for acute alcohol use and the odds of injury are concordant between the two control periods, which suggest that a 'hangover effect' is unlikely to bias our results. 'Hangover' effects generally start once BAC is close to zero [43-45] and this is less likely to influence results given we used a 6 h induction period.

Mis-reporting of alcohol use is another potential threat to the validity of this study [46]. If participants had improved memory of alcohol intake immediately before the cutting or piercing injury, compared to their control periods, then the effect of acute alcohol consumption on injury risk may have been inflated. A study investigating the causes of Meniere's disease, explored this phenomenon by repeated questioning of cases during attacks and in different control periods [46]. The authors concluded that outcome-dependent misclassification, was not a major threat to validity. No tendency to overestimate exposure close in time to attacks of the disease occurred, despite strong beliefs among patients of the likely causes of their acute symptoms. In addition, we did not ask if the person was at home using cutting tools in the control period which has been noted to be a potential bias in studies of motor vehicle injuries [47].

People who have higher socioeconomic status generally experience better health than those who are socially disadvantaged [48,49]. As well as being considered as potential confounders or effect modifiers in the relationship between alcohol and risk of injury, they are important parameters independently linked to injuries and, as such, need to be incorporated into injury prevention strategies and policy targeting reduction in home injuries. This study was a case-crossover study which is designed to examine transient risk factors and the strength of this design is that participants are their own controls and so cases are self-matched on socioeconomic factors. However, as a result of the study design, we cannot examine the influence of time-invariant exposures such as social status. In a related case-control study [5] we were able to explore these factors in the subset of our cases that had a landline and we found that the proportion of cases with no individual level socioeconomic deprivation characteristics (55.9%) was similar to that estimated for New Zealand adults (50.7%) [50]. The proportion of cases identifying as Māori or Pacific ethnicity (18.0% and 12.0% respectively) were higher than the expected proportions (9.7% and 9.0% respectively) based on 2006 Census figures for those in this age group and resident in the study regions [51].

Our findings contribute to the limited body of published evidence for risk factors associated with cutting or piercing injuries. The findings are consistent with previous research which has examined the association between acute alcohol use and unintentional injury [13,52,53]. A meta-analysis of acute alcohol use and different classes of injury reported a per-drink (10 g pure alcohol) pooled-effect estimate for unintentional injuries (other than falls or motor vehicle accidents) of OR 1.32 (95% CI: 1.27, 1.36) [52]. This effect measure is similar to our adjusted odds ratio of consuming 1 to 3 units compared to none of 1.77 (95% CI: 0.84, 3.74).

Our study found that the effect of alcohol on injury was stronger among smokers compared to non-smokers. The interaction of alcohol and smoking on a number of outcomes including fire and traffic injury has been the subject of a systematic review by Taylor et al. [54]. The authors concluded that this interaction may increase risk for traffic and fire injury, but suggested future research is required to confirm the relationship. Tobacco use, has previously been linked to some injuries [55], and impaired impulse control has been observed in smokers [56], and alcohol consumers [10]. Other explanations offered to explain the increased risk of injury among tobacco smokers include: direct toxicity from nicotine or carbon monoxide; distraction associated with lighting or disposing of cigarettes; or associated medical conditions; such as cardiovascular disease, cataracts or cancer which may impair performance of tasks; during which, injuries may occur [57]. Further analytical studies are required to confirm if the interaction between smoking and alcohol and injury risk exists.

## Conclusion

Cutting and piercing injuries are a leading cause of home injuries among young and working aged-adults. As is the case with injuries resulting from motor-vehicle crashes and falls [6,7,12,13,36,52,58], acute-alcohol intake contributes to unintentional cutting or piercing injuries among young and middle-aged adults. Our analysis suggests that it may treble the odds. In people who smoke tobacco such an effect is increased, a finding that is worthy of further exploration. The study findings add to the impetus to enact policies which dissuade problem drinking and limit access to alcohol which will reduce the risk of injuries both at home and on the highway.

## List of abbreviations

AUDIT: Alcohol use disorders identification test; BAC: Blood alcohol concentration; CI: Confidence interval; OR: Odds ratio; RR: Relative risk.

## Competing interests

The authors declare that they have no competing interests.

## Authors' contributions

ST led the analysis and interpretation of the study findings, and writing of the manuscript. BK and SA contributed to the conception, design, implementation and management of the study, the interpretation of the study findings, and critically revised the draft manuscript. ER contributed to the conception and design of the study, and the interpretation of the study findings. RM helped design the study's analytic strategy and contributed to the analysis and the interpretation of the study findings. GSM critically revised the draft manuscript for important intellectual content. BK is guarantor.

## Pre-publication history

The pre-publication history for this paper can be accessed here:

http://www.biomedcentral.com/1471-2458/11/852/prepub
